# Feasibility of generating sagittal radiographs from coronal views using GAN-based deep learning framework in adolescent idiopathic scoliosis

**DOI:** 10.1186/s41747-025-00553-6

**Published:** 2025-01-29

**Authors:** Tito Bassani, Andrea Cina, Fabio Galbusera, Andrea Cazzato, Maria Elena Pellegrino, Domenico Albano, Luca Maria Sconfienza

**Affiliations:** 1 IRCCS Istituto Ortopedico Galeazzi, Milan, Italy; 2 Department of Teaching, Research and Development, Schulthess Clinic, Zurich, Switzerland; 3 Department of Health Sciences and Technology (D-HEST), ETH Zürich, Zurich, Switzerland; 4 Department of Biomedical, Surgical, and Dental Sciences, University of Milan, Milan, Italy; 5 Department of Biomedical Sciences for Health, University of Milan, Milan, Italy

**Keywords:** Adolescent, Artificial intelligence, Deep learning, Lordosis, Scoliosis

## Abstract

**Background:**

Minimizing radiation exposure is crucial in monitoring adolescent idiopathic scoliosis (AIS). Generative adversarial networks (GANs) have emerged as valuable tools being able to generate high-quality synthetic images. This study explores the use of GANs to generate synthetic sagittal radiographs from coronal views in AIS patients.

**Methods:**

A dataset of 3,935 AIS patients who underwent spine and pelvis radiographic examinations using the EOS system, which simultaneously acquires coronal and sagittal images, was analyzed. The dataset was divided into training-set (85%, *n* = 3,356) and test-set (15%, *n* = 579). GAN model was trained to generate sagittal images from coronal views, with real sagittal views as reference standard. To assess accuracy, 100 subjects from the test-set were randomly selected for manual measurement of lumbar lordosis (LL), sacral slope (SS), pelvic incidence (PI), and sagittal vertical axis (SVA) by two radiologists in both synthetic and real images.

**Results:**

Sixty-nine synthetic images were considered assessable. The intraclass correlation coefficient ranged 0.93–0.99 for measurements in real images, and from 0.83 to 0.88 for synthetic images. Correlations between parameters of real and synthetic images were 0.52 (LL), 0.17 (SS), 0.18 (PI), and 0.74 (SVA). Measurement errors showed minimal correlation with scoliosis severity. Mean ± standard deviation absolute errors were 7 ± 7° (LL), 9 ± 7° (SS), 9 ± 8° (PI), and 1.1 ± 0.8 cm (SVA).

**Conclusion:**

While the model generates sagittal images visually consistent with reference images, their quality is not sufficient for clinical parameter assessment, except for promising results in SVA.

**Relevance statement:**

AI can generate synthetic sagittal radiographs from coronal views to reduce radiation exposure in monitoring adolescent idiopathic scoliosis (AIS). However, while these synthetic images appear visually consistent with real ones, their quality remains insufficient for accurate clinical assessment.

**Key Points:**

- AI can be exploited to generate synthetic sagittal radiographs from coronal views.
- Dataset of 3,935 subjects was used to train and test AI-model; spinal parameters from synthetic and real images were compared.
- Synthetic images were visually consistent with real ones, but quality was generally insufficient for accurate clinical assessment.

**Graphical Abstract:**

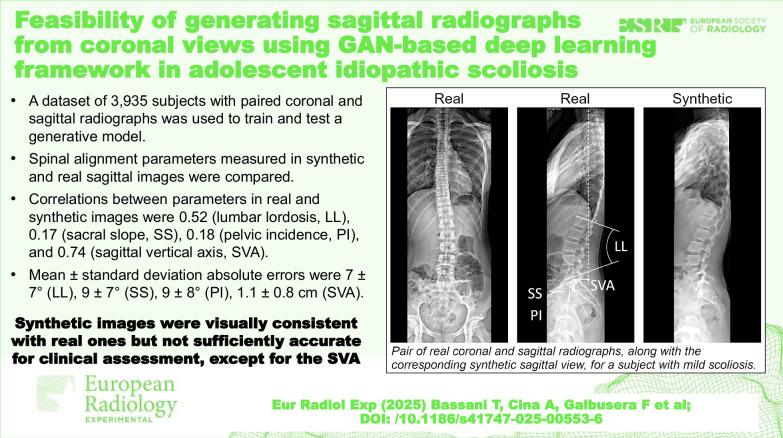

## Background

Adolescent idiopathic scoliosis (AIS) is a three-dimensional spinal deformity that manifests in children and adolescents, typically during their growth spurts. It is the most common type of scoliosis, accounting for approximately 80% of all scoliosis cases, and its causes remain unknown [1, 2]. Clinicians monitor AIS using radiographic imaging to assess the severity of spinal curvature and guide treatment decisions. While essential, this frequent imaging exposes patients to cumulative radiation, which poses long-term health risks, especially in young patients who are more sensitive to radiation effects. Given the necessity of repeated radiographs in AIS management, minimizing radiation exposure is a critical goal in clinical practice. In response to these concerns, low-dose radiography techniques have been developed and increasingly implemented in clinical settings [3–5]. However, even low-dose imaging carries some risks, and further reductions in radiation exposure would provide significant health benefits and cost reduction [6, 7]. To address these challenges, modern technological approaches, such as artificial intelligence, in particular machine learning, offer promising alternatives [8–10].

Generative deep learning models have emerged as powerful tools for image translation—the transformation of one type of image into another—in medical imaging [11–14]. Previous studies have focused, for example, on translating brain images between different modalities, such as computed tomography (CT), magnetic resonance imaging (MRI), and positron emission tomography [15–20], as well as between T1-weighted and T2-weighted MRI sequences [21, 22]. In spine imaging, preliminary applications have explored the generation of coronal and sagittal radiographs of the lumbar region [23], and the translation of sagittal images between MRI sequences [24] and between CT and MRI [25, 26]. Despite several limitations, these applications demonstrated the potential for transformative innovations in musculoskeletal radiology.

The deep learning models excel at learning complex patterns in imaging data and generating high-quality synthetic images. Among them, generative adversarial networks (GANs) [27–29] offer several advantages over alternative approaches, such as diffusion models [30–32], variational autoencoders [33, 34], and transformers [35, 36]. Specifically, GANs are designed for paired image-to-image translation tasks, making them particularly effective with annotated datasets to ensure high-quality mappings between input and target images. They are also renowned for producing sharper and more realistic images, owing to their adversarial training mechanism, which prioritizes the preservation of high-frequency details. Compared to diffusion models, GANs typically require fewer computational resources and achieve faster convergence. Additionally, their relative simplicity allows for easier implementation and tuning compared to the more complex frameworks of transformers, which often necessitate extensive pre-training and large-scale datasets. In recent years, the potential of GAN models to create realistic medical images has gained considerable attention, as they could reduce or eliminate the need for repeat imaging, thus minimizing radiation exposure [23, 24, 37–41]. These models can leverage deep learning to transform one imaging modality into another, such as generating synthetic radiographs from alternative views, highlighting their utility in advancing radiological workflows.

The present study investigates the application of GANs to generate synthetic sagittal radiographic images from coronal views in AIS patients. By utilizing a retrospective dataset of 3,935 subjects, we aim to investigate the feasibility and effectiveness of this approach. This methodology could offer a radiation-free alternative to sagittal radiographic imaging, reducing the need for additional exposures while maintaining clinical utility in monitoring spinal deformities.

## Methods

### Dataset

**Fig. 1:**
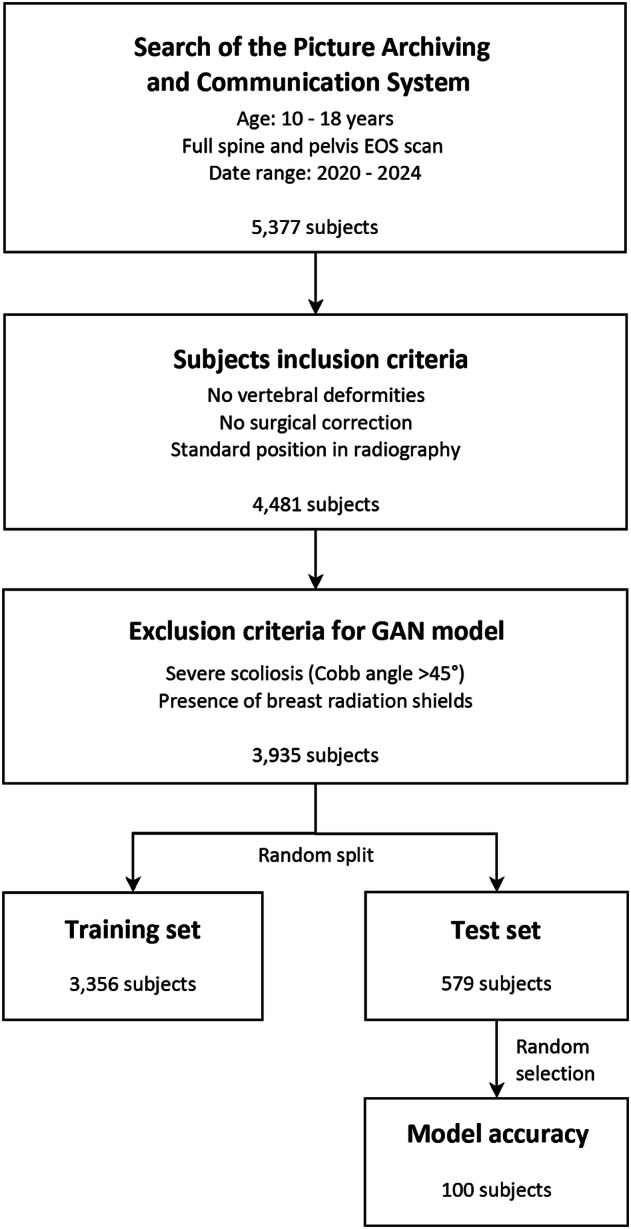
Chart diagram illustrating the workflow for the image selection

**Fig. 2:**
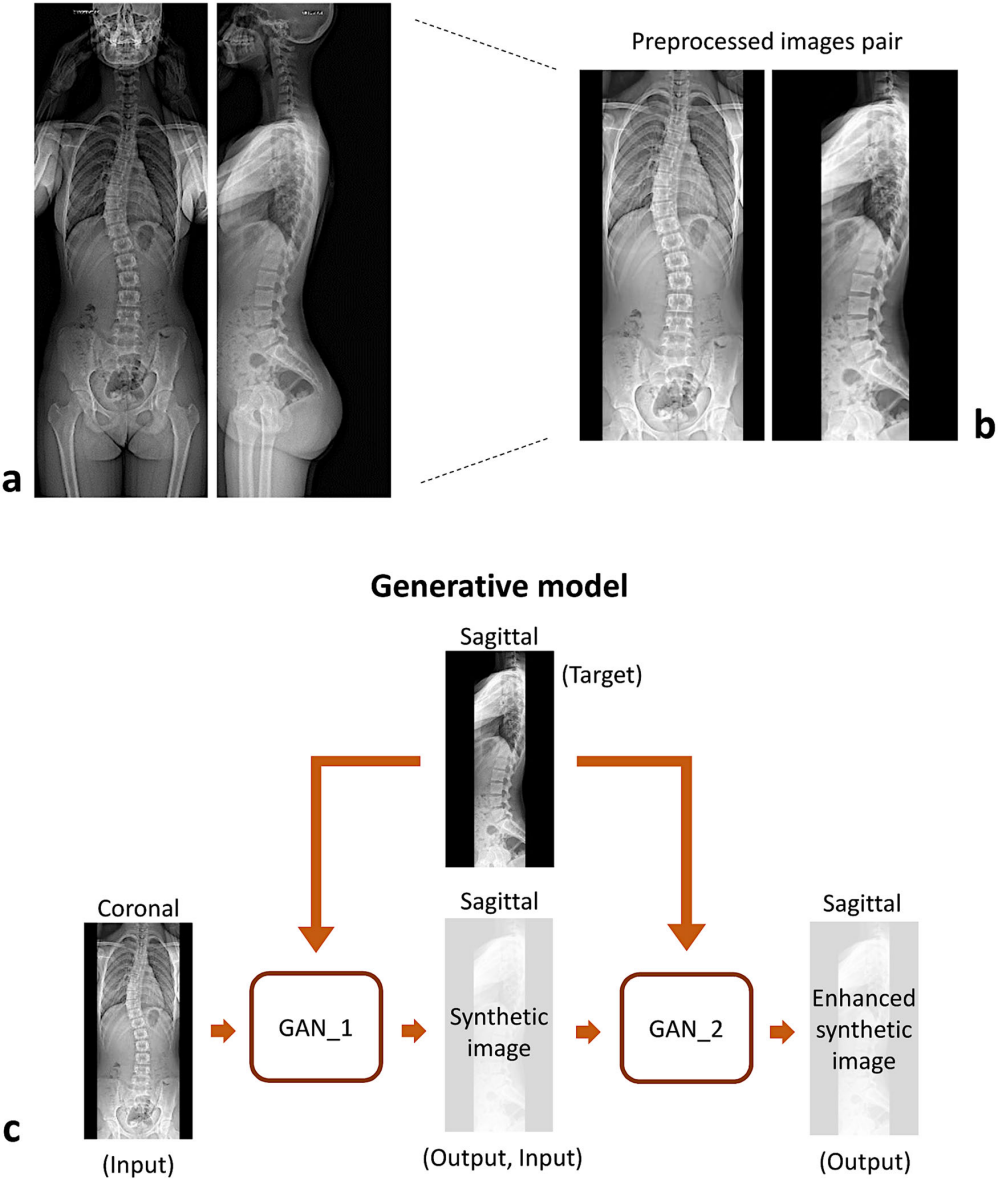
Upper panel: coronal and sagittal images pair acquired by EOS system (**a**) and pre-processed for generative adversarial network (GAN) model (**b**). Lower panel: GANs modeling scheme (**c**)

A retrospective search of the Picture Archiving and Communication System of the IRCCS Ospedale Galeazzi-Sant’Ambrogio (Milan, Italy) was performed on anonymized data acquired in the period 2020–2024 (Fig. 1). Subjects with the following criteria were included: age ranging from 10 to 18 years; radiographic examination of the spine and pelvis acquired by the EOS system (EOS Imaging, Paris, France), allowing for the simultaneous acquisition of true size coronal and sagittal images in one-to-one scale avoiding vertical distortion (Fig. 2a) [3–5]. Subjects with vertebral deformities or who underwent operative correction were excluded, as well as those presenting non-standard positions in biplanar radiography. A dataset of 4,481 subjects was obtained. The largest Cobb angle in the coronal plane, quantifying the scoliosis severity, was measured by a pool of four experienced radiologists. Images of subjects with severe scoliosis (Cobb angle greater than 45°) were excluded to remove cases with extreme spinal misalignments, along with those containing breast radiation shields (which could not be standardized by the generative model due to variations in presence, placement, and size). This yielded a final dataset of 3,935 subjects, which was randomly split into a training-set of 3,356 cases (85%) and a test-set of 579 cases (15%) (Fig. 1).

### Deep learning model

The preprocessing of the radiographic images involved the following steps: (i) bounding boxes encompassing the spine and pelvis, from the upper endplate of C7 to the femoral heads, were manually defined in both the coronal and sagittal planes by two experienced operators using an in-house application developed in MATLAB software (v.R2024b, MathWorks Inc., Natick, MA). For the coronal images, vertical and lateral limits were manually selected. Since the coronal and sagittal images acquired with the EOS system share the same vertical dimensions, the vertical limits defined in the coronal plane were applied to the sagittal plane. In contrast, the lateral limits in the sagittal plane were manually determined to include the femoral heads and the spine from C7 to the sacrum; (ii) the bounding box coordinates were used to crop the images; (iii) the cropped images were rescaled to 1,024 × 512 pixels, with black padding added to fill the image width; (iv) contrast was enhanced for both images by saturating the bottom 1% and top 1% of pixel values (Fig. 2b).

GAN model based on the pix2pix framework [28, 42] was employed to generate sagittal images from coronal ones. Two GANs were trained sequentially using the training-set (Fig. 2c). The first GAN (GAN_1) used real coronal images as input and real sagittal images as target outputs. The synthetic sagittal images generated by GAN_1 were then used as input for a second network (GAN_2), which was trained to enhance image resolution and contrast using real sagittal images as target outputs. The two GANs were trained separately and did not share any loss function information. Both GANs employed a U-Net architecture for the generator and a PatchGAN with a patch size of 256 pixels for the discriminator. The loss function used for training was a mean-squared error. Training was conducted with a batch size of 18 over 100 epochs. These hyperparameters were chosen as they provided the best accuracy in generating synthetic images while remaining within the upper limits of the available computational resources (Nvidia Quadro RTX 5000, 16 GB RAM GPU). All routines for image resizing and the implementation of the deep learning model were run in Python v.3.9, using the OpenCV library v. 4.8 (https://opencv.org/) and the TensorFlow Keras framework v.2.14 [43].

### Model accuracy and statistical analysis

Following the preliminary visual evaluation of synthetically generated images in the test-set, which indicated that not all images were potentially assessable for measuring sagittal parameters, a random subset of 100 cases was selected. These cases were manually evaluated for specific anatomical parameters in the sagittal plane by two radiologists with 10 and 15 years of experience. This approach aimed to reduce the workload for the two raters while ensuring that the selected subset represented an unbiased proportion of both assessable and nonassessable images, thus maintaining the integrity of the analysis. The radiologists independently evaluated the synthetic images, categorizing them as either assessable (*i.e*., where all the anatomical landmarks required for measuring the sagittal parameters were visible) or nonassessable. Only the cases categorized as assessable by both radiologists, without any mutual consultation, were included in the evaluation. The interobserver reliability of the classification was assessed using Cohen *κ* coefficient. The difference in the mean Cobb angle between assessable and nonassessable cases was analyzed using unpaired *t*-test in case of normal/near-normal distribution or a Wilcoxon rank-sum test in case of non-normal distribution.

**Fig. 3:**
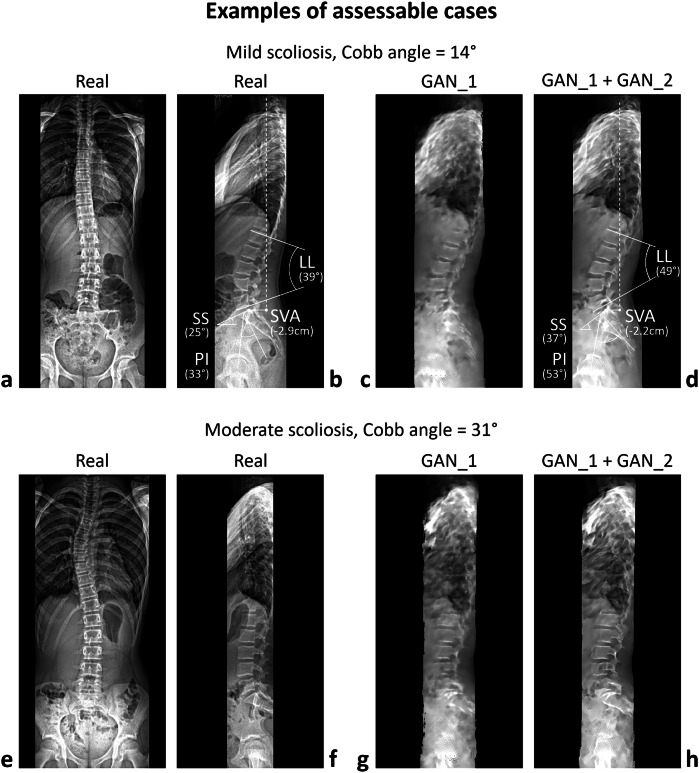
Examples of assessable cases, showing pairs of real coronal and sagittal images alongside the corresponding synthetic sagittal images generated by GAN_1 and GAN_1 + GAN_2 for patients with mild (**a**–**d**) and moderate (**e**–**h**) scoliosis. Measured sagittal parameters: lumbar lordosis (LL), pelvic incidence (PI), sacral slope (SS), and sagittal vertical axis (SVA)

For the assessable cases, the radiologists measured the following parameters on both synthetic and real images: lumbar lordosis (LL), defined as the angle between the line connecting the upper endplate corners of L1 and the line connecting the lower endplate corners of L5; sacral slope (SS), defined as the angle between the line connecting the corners of the sacral plate and the horizontal reference line; pelvic incidence (PI), defined as the angle between the line perpendicular to the sacral plate at its midpoint and the line connecting this point to the center of the bicoxofemoral axis; and sagittal vertical axis (SVA), defined as the horizontal distance between the plumb line dropped from the centroid of the C7 vertebra and the posterior corner of the sacral endplate, where a positive value indicates the line falls anterior to the posterior corner of the sacral endplate (Fig. 3b, d). The radiologists were not blinded to whether the images being assessed were real or synthetic, as the two types of images were easily distinguishable. Consequently, the two groups of images were evaluated separately, with all synthetic images assessed first, followed by the real images. To minimize potential bias related to recognizing similarities between subjects across groups, the images within each group were shuffled prior to evaluation. Only cases with synthetic images deemed assessable by both radiologists were included in the comparisons. The interobserver reliability of parameter measurements in both real and synthetic images was evaluated using the intraclass correlation coefficient [44].

Since each parameter was measured twice (once by each observer) for both real and synthetic images, the average measurement between observers was calculated for each image type. For each parameter, the association between the average measurements in real and synthetic images was assessed using the mean absolute error (*i.e*., the difference between the parameter values in real and synthetic images) and correlation analysis. The correlation coefficient, denoted as *r*, was calculated using either Pearson or Spearman rank coefficient, depending on whether a normal distribution was achieved. A value of 0 indicates no linear correlation, while -1 and 1 represent complete negative and positive correlations, respectively. The correlation coefficient between the measurement error (real *minus* synthetic) and the Cobb angle was also evaluated. To determine the statistical significance of the coefficients, a two-tailed *t*-test was used for the Pearson correlation, and a permutation distribution test was used for the Spearman coefficient. All the tests accounted for 0.05 as the significance *α* level. The analyses were performed in MATLAB software.

## Results

A total of 3,935 subjects (2,281 females, 58%; 1,654 males, 42%), aged between 10 and 18 years (mean SD: (14 ± 2 years, mean ± standard deviation) and with a Cobb angle of 17° ± 10°, were evaluated. Based on clinical practice, the subjects were classified into three categories: “non-scoliotic” (Cobb angle < 10°), “mild scoliosis” (Cobb angle 10°–25°), and “moderate scoliosis” (Cobb angle 25°–45°) (Table 1). The majority of cases fell into the mild scoliosis category (55%), with a higher prevalence of female subjects in both the mild and moderate scoliosis groups.

**Table 1:** Demographic data and Cobb angle in the considered dataset, for non-scoliotic subjects and cases with mild and moderate scoliosis

	Non-scoliotic	Mild	Moderate
Subjects (*n* = 3,935)	1,102 (28%)	2,164 (55%)	669 (17%)
Age (years)	13 ± 2	14 ± 2	15 ± 2
Sex (males/females)	590/512	669/1,298	315/551
Cobb angle (°)	6 ± 3	17 ± 5	32 ± 6

Values are given as a number of individuals and percentage or mean ± standard deviation

**Fig. 4:**
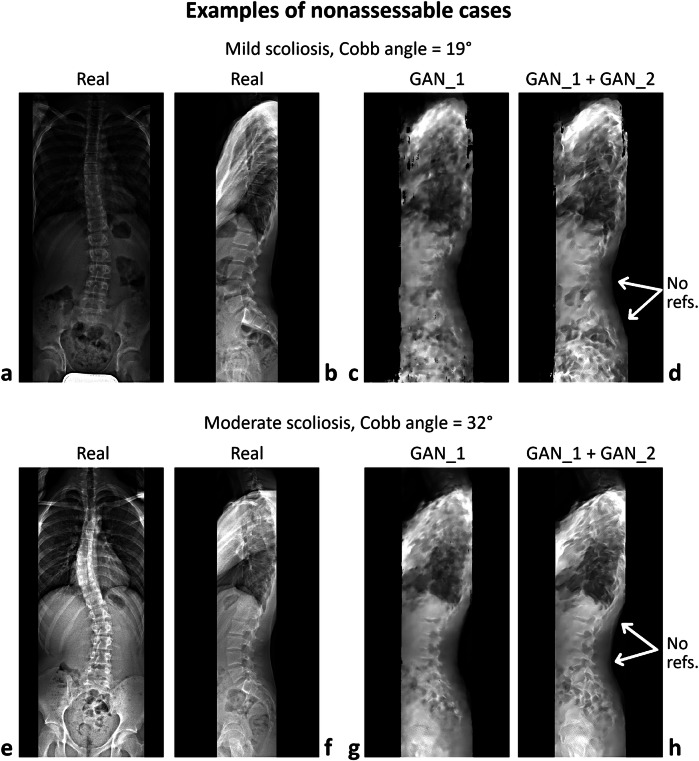
Examples of nonassessable cases, showing pairs of real coronal and sagittal images alongside the corresponding synthetic sagittal images generated by GAN_1 and GAN_1 + GAN_2 for patients with mild (**a**–**d**) and moderate (**e**–**h**) scoliosis. The white arrows in panels (**d**) and (**h**) highlight the absence of visible reference points (vertebral landmarks) necessary for the evaluation of sagittal parameters

**Fig. 5:**
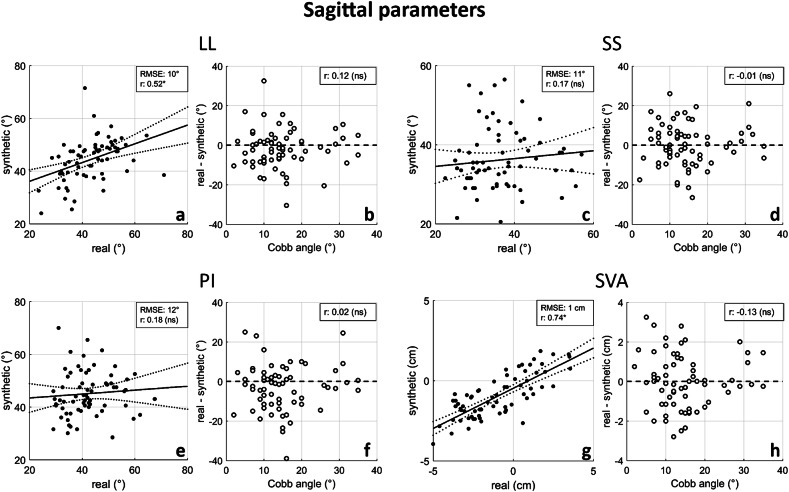
Scatter plots illustrating the relationship between parameter values in real and synthetic images, with regression lines, 95% confidence intervals, root mean square error (RMSE), and correlation coefficients (*r*, followed by an asterisk if significantly different from zero or “ns” if not significant) displayed in (**a**), (**c**), (**e**), and (**g**). **b**, **d**, **f**, and **h** depict the relationship between the Cobb angle and the measurement error in parameter values between real and synthetic images. Sagittal parameters: lumbar lordosis (LL), pelvic incidence (PI), sacral slope (SS), and sagittal vertical axis (SVA)

Of the 100 synthetic sagittal images selected for assessing model accuracy, only those categorized as assessable by both radiologists, without any mutual consultation, were included in the evaluation. Images were classified as assessable (see examples of mild and moderate scoliosis in Fig. 3) if all reference landmarks required for measuring sagittal parameters were visible; otherwise, they were categorized as nonassessable (examples in Fig. 4). Specifically, the two observers, with 10 and 15 years of experience, classified 22 and 29 cases as nonassessable, respectively, with 20 cases classified as nonassessable by both observers. The union of these classifications led to the exclusion of 31 subjects, resulting in a total of 69 assessable cases for evaluation. The interobserver κ coefficient for classification was 0.71 (95% confidence interval 0.55–0.87). The mean Cobb angle of nonassessable cases was significantly slightly larger than that of assessable ones (21° and 16°, respectively) (Table 2). The interobserver intraclass correlation coefficients for sagittal parameter measurements ranged from 0.93 to 0.99 for real sagittal images and from 0.83 to 0.88 for synthetic images (Table 3). The correlation between parameter values in real and synthetic images was 0.52 for LL, 0.17 for SS, 0.18 for PI, and 0.74 for SVA (Fig. 5a, c, e, g). The correlation between measurement error (*i.e*., the difference between measurements in real and synthetic images) and scoliosis severity, as measured by the Cobb angle, was negligible (Fig. 5b, d, f, h). The mean ± standard deviation absolute errors were 7 ± 7° for LL, 9 ± 7° for SS, 9 ± 8° for PI, and 1.1 ± 0.8 cm for SVA (Table 4). The largest absolute errors were 33° for LL, 27° for SS, 39° for PI, and 3.3 cm for SVA.

**Table 2:** Number of assessable and nonassessable synthetic images, and maximum Cobb angle in the corresponding group

	Assessable	Nonassessable
Number of cases	69	31
Cobb angle (°)	16 ± 10	21 ± 9*

Values are given as the number of individuals and percentage or mean ± standard deviation. * significant difference between groups (*p* = 0.007)

**Table 3:** Intraclass correlation coefficient (95% confidence interval) for interobserver agreement of sagittal parameters in real and synthetic images

	Lumbar lordosis	Sacral slope	Pelvic incidence	Sagittal vertical axis
Real images	0.95 (0.91–0.97)^a^	0.93 (0.89–0.96)^a^	0.94 (0.9–0.96)^a^	0.99 (0.98–0.99)^a^
Synthetic images	0.83 (0.74–0.89)^a^	0.88 (0.81–0.92)^a^	0.87 (0.8–0.92)^a^	0.88 (0.81–0.92)^a^

^a^ Coefficient significantly different from zero

## Discussion

This study investigated the application of GANs to synthesize sagittal radiographic images from coronal views in AIS patients, with the goal of showing the potential of reducing the need for sagittal imaging acquisition and its associated radiation exposure. The results demonstrate that the GAN-based approach can generate visually consistent synthetic sagittal images in most cases, showing strong interobserver reliability, although generally weak to moderate correlations were observed between real and synthetic images for key spinal measured parameters. This highlights several limitations that must be addressed before this technology can be reliably integrated into clinical practice.

A key outcome of the study is the high interobserver reliability for sagittal parameter measurements in synthetic images, reflected by intraclass correlation coefficients ranging from 0.83 to 0.88 (see Table 3). Although these values are slightly lower than those for real images (0.93 to 0.99), they still demonstrate substantial agreement between observers. This indicates that the synthetic images generated by the deep learning model are sufficiently consistent to allow for reproducible measurements by trained radiologists. However, it is important to note that the interobserver reliability in classifying cases as assessable or nonassessable was moderate-good (0.71), with the more experienced radiologist being more stringent (29 *versus* 22 cases classified as nonassessable). This variability limits the potential clinical impact of the generative model in terms of evaluation agreement.

Overall, the relatively high interobserver agreement for the sagittal parameters measured on synthetic images can be attributed to the advanced architecture of the GAN model employed in the study, which uses a U-Net generator and PatchGAN discriminator to capture both fine anatomical details and global image features. Moreover, the sequential training of two GANs—one focused on generating synthetic images and the other on enhancing their resolution and contrast—likely contributed to the improved image quality and consistency (see Fig. 2c). Notably, the enhancement in resolution achieved by applying GAN_2 to the synthetic images generated by GAN_1, although moderate, was not negligible (see Figs. 3 and 4). This improvement facilitated the identification of vertebral landmarks necessary for the measurement of sagittal parameters.

The correlations between real and synthetic images for key sagittal parameters provide insights into the potential clinical utility of the generated images. Notably, SVA showed a correlation of 0.74 (see Fig. 5g), indicating a strong positive relationship between the measurements in real and synthetic images. Given that SVA is a critical parameter in assessing spinal alignment and surgical planning for AIS patients, this result is particularly promising. However, the correlations for LL, SS, and PI were notably lower, ranging only from 0.17 to 0.52 (see Fig. 5a, c, e). These weaker correlations suggest that while the GAN model is effective in replicating certain aspects of sagittal anatomy, its ability to accurately capture more complex or subtle spinal alignments remains limited.

The absolute errors for the parameters between real and synthetic images were larger than those typically expected from human operators in a clinical context [45–47], with mean ± standard deviation values ranging from 7 ± 7° to 9 ± 8° for LL, SS, and PI and up to 1.1 ± 0.8 cm for SVA (see Table 4). Moreover, the largest errors observed (33° for LL, 27° for SS, 39° for PI, and 3.3 cm for SVA) underscore the challenges in generating highly accurate synthetic images. Despite these discrepancies, the impact of spinal deformity severity on synthetic image generation appeared to be generally weak. Although the assessable cases exhibited moderately lower mean Cobb angle compared to nonassessable ones (16° *versus* 21°, respectively) (see Table 2), measurement errors in assessable cases showed poor correlation with scoliosis severity (see Fig. 5b, d, f, h). Specifically, the distribution of results was uniformly scattered across the entire range of Cobb angles, indicating similar trends for non-scoliotic cases as well as those with mild or moderate scoliosis. In other words, while it appears slightly more challenging for the deep learning model to generate assessable images (*i.e*., those with all anatomical landmarks visible) for cases of more severe scoliosis, the error in landmark positioning is not directly associated with scoliosis severity. These findings suggest that the difficulty of the GAN model in generating clinically relevant sagittal images is not strongly influenced by the degree of spinal deformity in the coronal plane. However, the exclusion of patients with severe scoliosis (Cobb angle larger than 45°) from the study limits the generalizability of this conclusion. Further research is needed to gain a deeper understanding of the dependence of model performance on scoliosis severity and to evaluate the performance in more severe cases, where accurate assessment of sagittal parameters is often more challenging and clinically important.

**Table 4:** Absolute error between measurements of sagittal parameters in real and synthetic images

	Lumbar lordosis	Sacral slope	Pelvic incidence	Sagittal vertical axis
Absolute error	7 ± 7 (0–33)°	9 ± 7 (0–27)°	9 ± 8 (0–39)°	1.1 ± 0.8 (0–3.3) cm

Values are given as mean ± standard deviation (range)

**Fig. 6:**
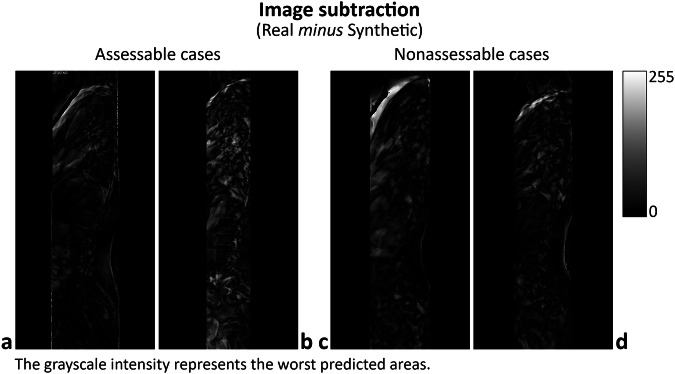
Image subtraction, real *minus* synthetic (from GAN_1 + GAN_2), for the assessable cases (**a**, **b**) shown in Fig. 3 and nonassessable cases (**c**, **d**) presented in Fig. 4. The images are rendered in grayscale, with intensity representing the absolute difference in pixel values between the real and synthetic images. Pixel intensity ranges from 0 (black) to 255 (white), representing the maximum difference obtained from the subtraction of grayscale images encoded as unsigned 8-bit

Several factors may contribute to the low accuracy in the measurement of LL, SS, and PI. First, these parameters rely heavily on the precise identification of anatomical landmarks, which can be more difficult to accurately reconstruct in synthetic images compared to real radiographs (see Fig. 3 and 4). While the GAN model was trained on a large dataset of real images, the inherent variability in lumbar spine curvature and pelvic anatomy across AIS patients may introduce discrepancies in the generated images. Additionally, the relatively low resolution of the input coronal images, despite being rescaled and pre-processed, may have limited the model’s ability to capture finer anatomical details necessary for accurately measuring these parameters. This observation was confirmed by the evaluation of image subtraction, a technique that generates a visual representation by subtracting the numerical pixel values of the predicted image from those of the real image. This process highlights regions with higher and lower prediction accuracy, indicated by the absolute value of the differences. Subtracting the synthetic image (produced by GAN_1 + GAN_2) from the real image generally demonstrated similar performance across the resulting image, irrespective of whether the cases were assessable or nonassessable (Fig. 6). Image subtraction also demonstrated a decline in model accuracy in the presence of potentially disruptive elements, such as superimposed air in the coronal input image. Cases with air in the abdominal region (darker areas in Fig. 3a, e and Fig. 4a) exhibited poorer predictions in the corresponding regions of the subtracted image (lighter areas anterior to the lumbar spine in Fig. 6a–c) compared to cases without air (Figs. 4e and  6d). However, this effect did not appear to impact the generation of vertebral landmarks. This may be attributed to the reliance on the positioning of vertebral bones in the coronal image, which is centrally located, whereas air is typically situated laterally to the spine in the abdominal region. Overall, future studies could address these issues by incorporating higher-resolution input images and refining the GAN architecture to improve landmark detection.

In addition, the overall accuracy in measuring sagittal parameters is likely worse than reported, as 31% of the synthetic images were deemed nonassessable and excluded from quantitative analysis. These excluded images clearly failed to meet clinical expectations. Notably, this study represents the first exploration of the feasibility of translating coronal spine radiographs into sagittal views in AIS. The decision to employ the pix2pix GAN model was driven by its advantages in terms of computational efficiency, ease of implementation, and tuning compared to alternative approaches such as diffusion models, variational autoencoders, and transformers. Future studies should consider comparing these results with those obtained using alternative methods to provide a more comprehensive evaluation. Regarding the model architecture, the image input size for the U-Net generator was relatively large (1,024 × 512 pixels), which leads to faster saturation of computational resources when attempting to increase the batch size during model training, potentially affecting model performance. The use of random input patches was initially considered as a strategy to reduce the image input size. However, this approach was not adopted, as reconstructing the entire sagittal image from a set of subregion images generated from the patches would compromise coherence in terms of the number of vertebrae and their relative positioning. Such coherence is essential for accurately identifying vertebral levels, which is critical for the proper measurement of sagittal parameters. For instance, LL requires precise identification of L1 and L5, while SVA depends on the correct positioning of C7. The use of smaller input sizes remains limited and may be suitable for generating subregion images (*e.g*., lumbar or lumbo-pelvic regions) to focus on measuring specific spinal parameters.

Data augmentation was excluded during model training for several reasons. First, augmentation techniques such as rotations, translations, or scaling can distort anatomical structures, resulting in unrealistic or anatomically incorrect outputs that compromise the identification of specific landmarks or features (*e.g*., vertebral alignment). Second, with large image sizes, maintaining spatial coherence across the entire image becomes critical, and data augmentation could disrupt spatial relationships, particularly when reconstructing complex anatomical regions such as the spine. Third, in our study, the GAN was trained to perform a specific transformation from coronal to sagittal views. Introducing augmented variations could shift the data distribution, making it more difficult for the GAN to learn the one-to-one mapping between the input (coronal) and output (sagittal) views. Finally, augmented features might not align with clinically relevant scenarios. For instance, flipping or rotating an image may not correspond to realistic variations in anatomical presentations, potentially leading the model to learn features that are irrelevant or even detrimental to the task.

Regarding potential improvements for future research, it is important to note that incorporating additional imaging modalities, such as CT or MRI, to provide complementary information may not be practical. CT imaging involves increased radiation exposure, which is particularly unsuitable for adolescent subjects. Similarly, traditional MRI, typically acquired in the supine position, introduces significant discrepancies in spinal alignment compared to standing position x-rays. Instead, advancements in model architecture, such as attention mechanisms, progressive growing, or hybrid models (*e.g*., conditional and cycle GANs), could offer promising avenues for improvement. Additional subject information such as height and weight, which were not available in the study because it was not included in the searched archive, could be accounted for as conditional input and also offer an evaluation of the potential relationship between model accuracy and subject characteristics such as body mass index.

In conclusion, this study demonstrates the feasibility of using GANs to generate synthetic sagittal radiographs from coronal images in AIS patients, with promising results in terms of interobserver reliability. However, while the model generates sagittal images visually consistent with reference images, their quality is not sufficient for clinical parameter assessment, except for the promising results seen in SVA, which describes the lateral plumb line alignment. While challenges remain in accurately modeling spinal anatomy, the potential for reducing radiation exposure through the use of synthetic images represents an exciting development in the management of AIS. Further research and refinement of the model are needed to fully realize its clinical potential.

## Data Availability

The datasets used and/or analyzed during the current study are available from the corresponding author upon reasonable request.

## Abbreviations

AIS: Adolescent idiopathic scoliosis
CT: Computed tomography
GAN: Generative adversarial network
LL: Lumbar lordosis
PI: Pelvic incidence
SS: Sacral slope
SVA: Sagittal vertical axis

## References

[1] Weinstein SL, Dolan LA, Cheng JC, Danielsson A, Morcuende JA (2008) Adolescent idiopathic scoliosis. Lancet 371:1527–1537. 10.1016/S0140-6736(08)60658-3 18456103 10.1016/S0140-6736(08)60658-3

[2] Nnadi C, Fairbank J (2010) Scoliosis: a review. Paediatrics Child Health 20:215–220. 10.1016/j.paed.2009.11.009

[3] Dietrich TJ, Pfirrmann CW, Schwab A, Pankalla K, Buck FM (2013) Comparison of radiation dose, workflow, patient comfort and financial break-even of standard digital radiography and a novel biplanar low-dose X-ray system for upright full-length lower limb and whole spine radiography. Skelet Radiol 42:959–967. 10.1007/s00256-013-1600-0 10.1007/s00256-013-1600-0 23536038

[4] Somoskeoy S, Tunyogi-Csapo M, Bogyo C, Illes T (2012) Accuracy and reliability of coronal and sagittal spinal curvature data based on patient-specific three-dimensional models created by the EOS 2D/3D imaging system. Spine J 12:1052–1059. 10.1016/j.spinee.2012.10.002 23102842 10.1016/j.spinee.2012.10.002

[5] Illes T, Somoskeoy S (2012) The EOS imaging system and its uses in daily orthopaedic practice. Int Orthop 36:1325–1331. 10.1007/s00264-012-1512-y 22371113 10.1007/s00264-012-1512-y PMC3385897

[6] Negrini S, Donzelli S, Aulisa AG et al (2018) 2016 SOSORT guidelines: Orthopaedic and rehabilitation treatment of idiopathic scoliosis during growth. Scoliosis Spinal Disord 13:3–8. 10.1186/s13013-017-0145-8. eCollection 2018 10.1186/s13013-017-0145-8 PMC5795289 29435499

[7] Tomaiuolo R, Banfi G, Messina C, Albano D, Gitto S, Sconfienza LM (2024) Health technology assessment in musculoskeletal radiology: the case study of EOSedge™. Radiol Med 129:1076–1085. 10.1007/s11547-024-01832-9 38856961 10.1007/s11547-024-01832-9 PMC11252187

[8] Galbusera F, Casaroli G, Bassani T (2019) Artificial intelligence and machine learning in spine research. JOR Spine 2:e1044. 10.1002/jsp2.1044 31463458 10.1002/jsp2.1044 PMC6686793

[9] Hornung AL, Hornung CM, Mallow GM et al (2022) Artificial intelligence in spine care: current applications and future utility. Eur Spine J 31:2057–2081. 10.1007/s00586-022-07176-0 35347425 10.1007/s00586-022-07176-0

[10] Wang S, Cao G, Wang Y et al (2021) Review and prospect: Artificial intelligence in advanced medical imaging. Front Radiol 1:781868. 10.3389/fradi.2021.781868 37492170 10.3389/fradi.2021.781868 PMC10365109

[11] Sizikova E, Badal A, Delfino JG et al (2024) Synthetic data in radiological imaging: current state and future outlook. BJR Artif Intell 1:ubae007. 10.1093/bjrai/ubae007 10.1093/bjrai/ubae007 PMC13045695 42064396

[12] Jung HK, Kim K, Park JE, Kim N (2024) Image-based generative artificial intelligence in radiology: comprehensive updates. Korean J Radiol 25:959–981. 10.3348/kjr.2024.0392 39473088 10.3348/kjr.2024.0392 PMC11524689

[13] Chen J, Chen S, Wee L, Dekker A, Bermejo I (2023) Deep learning based unpaired image-to-image translation applications for medical physics: a systematic review. Phys Med Biol. 10.1088/1361-6560/acba74 10.1088/1361-6560/acba74 36753766

[14] Koetzier LR, Wu J, Mastrodicasa D et al (2024) Generating synthetic data for medical imaging. Radiology 312:e232471. 10.1148/radiol.232471 39254456 10.1148/radiol.232471 PMC11444329

[15] Abu-Srhan A, Almallahi I, Abushariah MAM, Mahafza W, Al-Kadi OS (2021) Paired-unpaired unsupervised attention guided GAN with transfer learning for bidirectional brain MR-CT synthesis. Comput Biol Med 136:104763. 10.1016/j.compbiomed.2021.104763 34449305 10.1016/j.compbiomed.2021.104763

[16] Pan S, Abouei E, Wynne J et al (2024) Synthetic CT generation from MRI using 3D transformer-based denoising diffusion model. Med Phys 51:2538–2548. 10.1002/mp.16847 38011588 10.1002/mp.16847 PMC10994752

[17] Xie T, Cao C, Cui Z et al (2023) Brain PET synthesis from MRI using joint probability distribution of diffusion model at ultrahigh fields. https://arxiv.org/abs/2211.08901

[18] Bahloul MA, Jabeen S, Benoumhani S, Alsaleh HA, Belkhatir Z, Al-Wabil A (2024) Advancements in synthetic CT generation from MRI: a review of techniques, and trends in radiation therapy planning. J Appl Clin Med Phys 25:e14499. 10.1002/acm2.14499 39325781 10.1002/acm2.14499 PMC11539972

[19] Fard AS, Reutens DC, Ramsay SC, Goodman SJ, Ghosh S, Vegh V (2024) Image synthesis of interictal SPECT from MRI and PET using machine learning. Front Neurol 15:1383773. 10.3389/fneur.2024.1383773 38988603 10.3389/fneur.2024.1383773 PMC11234346

[20] Lin W, Lin W, Chen G et al (2021) Bidirectional mapping of brain MRI and PET with 3D reversible GAN for the diagnosis of Alzheimer’s disease. Front Neurosci 15:646013. 10.3389/fnins.2021.646013 33935634 10.3389/fnins.2021.646013 PMC8080880

[21] Liu X, Xing F, Fakhri GE, Woo J (2021) A unified conditional disentanglement framework for multimodal brain mr image translation. Proc IEEE Int Symp Biomed Imaging 2021. 10.1109/isbi48211.2021.9433897 10.1109/isbi48211.2021.9433897 PMC8460116 34567419

[22] Kawahara D, Nagata Y (2021) T1-weighted and T2-weighted MRI image synthesis with convolutional generative adversarial networks. Rep Pract Oncol Radiother 26:35–42. 10.5603/RPOR.a2021.0005 33948300 10.5603/RPOR.a2021.0005 PMC8086713

[23] Galbusera F, Niemeyer F, Seyfried M et al (2018) Exploring the potential of generative adversarial networks for synthesizing radiological images of the spine to be used in in silico trials. Front Bioeng Biotechnol 6:53. 10.3389/fbioe.2018.00053 29780802 10.3389/fbioe.2018.00053 PMC5946008

[24] Galbusera F, Bassani T, Casaroli G et al (2018) Generative models: an upcoming innovation in musculoskeletal radiology? A preliminary test in spine imaging. Eur Radiol Exp 2:29–7. 10.1186/s41747-018-0060-7 30377873 10.1186/s41747-018-0060-7 PMC6207611

[25] Hong K, Cho Y, Kang CH et al (2022) Lumbar spine computed tomography to magnetic resonance imaging synthesis using generative adversarial network: visual turing test. Diagnostics (Basel) 12:530. 10.3390/diagnostics12020530 35204619 10.3390/diagnostics12020530 PMC8871227

[26] Roberts M, Hinton G, Wells AJ et al (2023) Imaging evaluation of a proposed 3D generative model for MRI to CT translation in the lumbar spine. Spine J 23:1602–1612. 10.1016/j.spinee.2023.06.399 37479140 10.1016/j.spinee.2023.06.399

[27] Singh NK, Raza K (2021) Medical image generation using generative adversarial networks: A review. In: Patgiri R, Biswas A, Roy P (eds). Health informatics: a computational perspective in healthcare. Springer Singapore, Singapore, pp. 77–96

[28] Zhou T, Li Q, Lu H, Cheng Q, Zhang X (2023) GAN review: models and medical image fusion applications. Inf Fusion 91:134–148. 10.1016/j.inffus.2022.10.017

[29] Armanious K, Jiang C, Fischer M et al (2020) MedGAN: medical image translation using GANs. Comput Med Imaging Graph 79:101684. 10.1016/j.compmedimag.2019.101684 31812132 10.1016/j.compmedimag.2019.101684

[30] Kazerouni A, Aghdam EK, Heidari M et al (2023) Diffusion models in medical imaging: a comprehensive survey. Med Image Anal 88:102846. 10.1016/j.media.2023.102846 37295311 10.1016/j.media.2023.102846

[31] Hung ALY, Zhao K, Zheng H et al (2023) Med-cDiff: conditional medical image generation with diffusion models. Bioengineering (Basel) 10:1258. 10.3390/bioengineering10111258 10.3390/bioengineering10111258 PMC10669033 38002382

[32] Zhou Y, Chen T, Hou J et al (2024) Cascaded multi-path shortcut diffusion model for medical image translation. Med Image Anal 98:103300. 10.1016/j.media.2024.103300 39226710 10.1016/j.media.2024.103300 PMC11979896

[33] Rais K, Amroune M, Benmachiche A, Haouam MY (2024) Exploring variational autoencoders for medical image generation: a comprehensive study. https://arxiv.org/abs/2411.07348

[34] Ehrhardt J, Wilms M (2022) Chapter 8—autoencoders and variational autoencoders in medical image analysis. In: Burgos N, Svoboda D (eds). Biomedical image synthesis and simulation. Academic Press, pp. 129–162

[35] Shamshad F, Khan S, Zamir SW et al (2023) Transformers in medical imaging: a survey. Med Image Anal 88:102802. 10.1016/j.media.2023.102802 37315483 10.1016/j.media.2023.102802

[36] Li J, Chen J, Tang Y, Wang C, Landman BA, Zhou SK (2023) Transforming medical imaging with transformers? A comparative review of key properties, current progresses, and future perspectives. Med Image Anal 85:102762. 10.1016/j.media.2023.102762 36738650 10.1016/j.media.2023.102762 PMC10010286

[37] Yoon JT, Lee KM, Oh J, Kim H, Jeong JW (2024) Insights and considerations in development and performance evaluation of generative adversarial networks (GANs): what radiologists need to know. Diagnostics (Basel) 14:1756. 10.3390/diagnostics14161756 39202244 10.3390/diagnostics14161756 PMC11353572

[38] Abbasi S, Lan H, Choupan J, Sheikh-Bahaei N, Pandey G, Varghese B (2024) Deep learning for the harmonization of structural MRI scans: a survey. Biomed Eng Online 23:90–96. 10.1186/s12938-024-01280-6 39217355 10.1186/s12938-024-01280-6 PMC11365220

[39] Vey BL, Gichoya JW, Prater A, Hawkins CM (2019) The role of generative adversarial networks in radiation reduction and artifact correction in medical imaging. J Am Coll Radiol 16:1273–1278. 10.1016/j.jacr.2019.05.040 31492405 10.1016/j.jacr.2019.05.040

[40] Koshino K, Werner RA, Pomper MG et al (2021) Narrative review of generative adversarial networks in medical and molecular imaging. Ann Transl Med 9:821–6325. 10.21037/atm-20-6325 34268434 10.21037/atm-20-6325 PMC8246192

[41] Han C, Hayashi H, Rundo L et al (2018) GAN-based synthetic brain MR image generation. 2018 IEEE 15th International Symposium on Biomedical Imaging (ISBI 2018):734–738. 10.1109/ISBI.2018.8363678

[42] Isola P, Zhu J, Zhou T, Efros AA (2018) Image-to-image translation with conditional adversarial networks. https://arxiv.org/abs/1611.07004

[43] Abadi M, Agarwal A, Barham P et al (2016) TensorFlow: Large-scale machine learning on heterogeneous distributed systems. https://arxiv.org/abs/1603.04467

[44] Koo TK, Li MY (2016) A guideline of selecting and reporting intraclass correlation coefficients for reliability research. J Chiropr Med 15:155–163. 10.1016/j.jcm.2016.02.012 27330520 10.1016/j.jcm.2016.02.012 PMC4913118

[45] Dang NR, Moreau MJ, Hill DL, Mahood JK, Raso J (2005) Intra-observer reproducibility and interobserver reliability of the radiographic parameters in the spinal deformity study group’s AIS radiographic measurement manual. Spine (Phila Pa 1976) 30:1064–1069. 10.1097/01.brs.0000160840.51621.6b 15864160 10.1097/01.brs.0000160840.51621.6b

[46] Dimar JR 2nd, Carreon LY, Labelle H et al (2008) Intra- and inter-observer reliability of determining radiographic sagittal parameters of the spine and pelvis using a manual and a computer-assisted methods. Eur Spine J 17:1373–1379. 10.1007/s00586-008-0755-1 18726124 10.1007/s00586-008-0755-1 PMC2556466

[47] Berthonnaud E, Labelle H, Roussouly P, Grimard G, Vaz G, Dimnet J (2005) A variability study of computerized sagittal spinopelvic radiologic measurements of trunk balance. J Spinal Disord Tech 18:66–71. 10.1097/01.bsd.0000128345.32521.43 15687855 10.1097/01.bsd.0000128345.32521.43

